# Structural basis for the regulation of enzymatic activity of Regnase-1 by domain-domain interactions

**DOI:** 10.1038/srep22324

**Published:** 2016-03-01

**Authors:** Mariko Yokogawa, Takashi Tsushima, Nobuo N. Noda, Hiroyuki Kumeta, Yoshiaki Enokizono, Kazuo Yamashita, Daron M. Standley, Osamu Takeuchi, Shizuo Akira, Fuyuhiko Inagaki

**Affiliations:** 1 Faculty of Advanced Life Science, Hokkaido University, Sapporo 001-0021, Japan; 2 Graduate school of Life Science, Hokkaido University, Sapporo 060-0810, Japan; 3 Institute of Microbial Chemistry, Microbial Chemistry Research Foundation, Tokyo 141-0021, Japan; 4 World Premier International Immunology Frontier Research Center, Osaka University, Osaka 565-0871, Japan; 5 Research Institute for Microbial Diseases, Osaka University, Osaka 565-0871, Japan; 6 Institute for Virus Research, Kyoto University, Kyoto 606-8507, Japan

## Abstract

Regnase-1 is an RNase that directly cleaves mRNAs of inflammatory genes such as IL-6 and IL-12p40, and negatively regulates cellular inflammatory responses. Here, we report the structures of four domains of Regnase-1 from *Mus musculus*—the N-terminal domain (NTD), PilT N-terminus like (PIN) domain, zinc finger (ZF) domain and C-terminal domain (CTD). The PIN domain harbors the RNase catalytic center; however, it is insufficient for enzymatic activity. We found that the NTD associates with the PIN domain and significantly enhances its RNase activity. The PIN domain forms a head-to-tail oligomer and the dimer interface overlaps with the NTD binding site. Interestingly, mutations blocking PIN oligomerization had no RNase activity, indicating that both oligomerization and NTD binding are crucial for RNase activity *in vitro*. These results suggest that Regnase-1 RNase activity is tightly controlled by both intramolecular (NTD-PIN) and intermolecular (PIN-PIN) interactions.

The initial sensing of infection is mediated by a set of pattern-recognition receptors (PRRs) such Toll-like receptors (TLRs) and the intracellular signaling cascades triggered by TLRs evoke transcriptional expression of inflammatory mediators that coordinate the elimination of pathogens and infected cells^1^,^2^,^3^. Since aberrant activation of this system leads to auto immune disorders, it must be tightly regulated. Regnase-1 (also known as Zc3h12a and MCPIP1) is an RNase whose expression level is stimulated by lipopolysaccharides and prevents autoimmune diseases by directly controlling the stability of mRNAs of inflammatory genes such as interleukin (IL)-6, IL-1β, IL-2, and IL-12p40^4^,^5^,^6^,^7^. Regnase-1 accelerates target mRNA degradation via their 3′-terminal untranslated region (3′UTR), and also degrades its own mRNA^8^.

Regnase-1 is a member of Regnase family and is composed of a PilT N-terminus like (PIN) domain followed by a CCCH-type zinc–finger (ZF) domain, which are conserved among Regnase family members^7^,^9^,^10^. Recently, the crystal structure of the Regnase-1 PIN domain derived from *Homo sapiens* was reported^11^. The structure combined with functional analyses revealed that four catalytically important Asp residues form the catalytic center and stabilize Mg^2+^ binding that is crucial for RNase activity. Several CCCH-type ZF motifs in RNA-binding proteins have been reported to directly bind RNA^12^,^13^,^14^,^15^. In addition, Regnase-1 has been predicted to possess other domains in the N- and C- terminal regions^16^,^17^. However, the structure and function of the ZF domain, N-terminal domain (NTD) and C-terminal domain (CTD) of Regnase-1 have not been solved.

Here, we performed structural and functional analyses of individual domains of Regnase-1 derived from *Mus musculus* in order to understand the catalytic activity *in vitr*o. Our data revealed that the catalytic activity of Regnase-1 is regulated through both intra and intermolecular domain interactions *in vitr*o. The NTD plays a crucial role in efficient cleavage of target mRNA, through intramolecular NTD-PIN interactions. Moreover, Regnase-1 functions as a dimer through intermolecular PIN-PIN interactions during cleavage of target mRNA. Our findings suggest that Regnase-1 cleaves its target mRNA by an NTD-activated functional PIN dimer, while the ZF increases RNA affinity in the vicinity of the PIN dimer.

## Results

### Domain structures of Regnase-1

**Figure 1:**
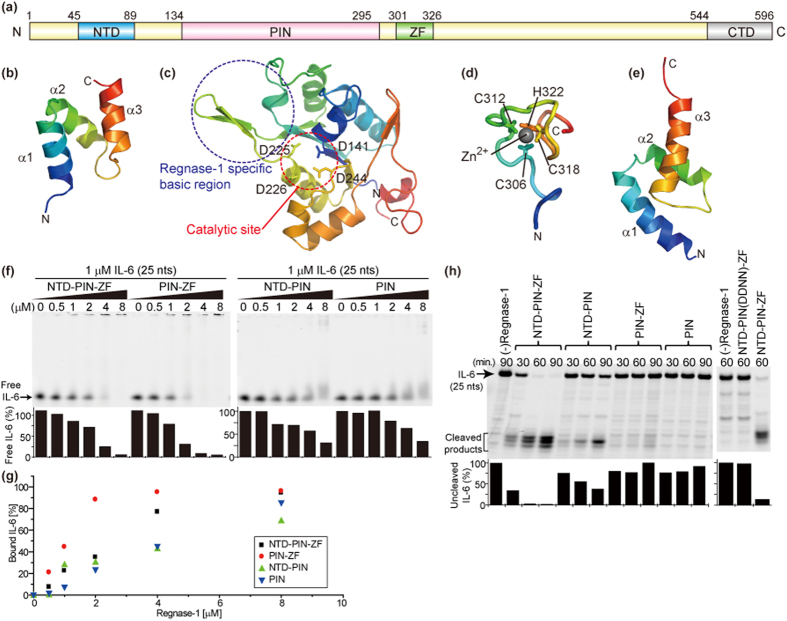
Structural and functional analyses of Regnase-1. (**a**) Domain architecture of Regnase-1. (**b**) Solution structure of the NTD. (**c**) Crystal structure of the PIN domain. Catalytic Asp residues were shown in sticks. (**d**) Solution structure of the ZF domain. Three Cys residues and one His residue responsible for Zn^2+^-binding were shown in sticks. (**e**) Solution structure of the CTD. All the structures were colored in rainbow from N-terminus (blue) to C-terminus (red). (**f**) *In vitro* gel shift binding assay between Regnase-1 and IL-6 mRNA. Fluorescence intensity of the free IL-6 in each sample was indicated as the percentage against that in the absence of Regnase-1. (**g**) Binding of Regnase-1 and IL-6 mRNA was plotted. The percentage of the bound IL-6 was calculated based on the fluorescence intensities of the free IL-6 quantified in (**f**). (**h**) *In vitro* cleavage assay of Regnase-1 to IL-6 mRNA. Fluorescence intensity of the uncleaved IL-6 mRNA was indicated as the percentage against that in the absence of Regnase-1.

We analyzed Rengase-1 derived from *Mus musculus* and solved the structures of the four domains; NTD, PIN, ZF, and CTD individually by X-ray crystallography or NMR (Fig. 1a–e). X-ray crystallography was attempted for the fragment containing both the PIN and ZF domains, however, electron density was observed only for the PIN domain (Fig. 1c), consistent with a previous report on Regnase-1 derived from *Homo sapiens*^11^. This suggests that the PIN and ZF domains exist independently without interacting with each other. The domain structures of NTD, ZF, and CTD were determined by NMR (Fig. 1b,d,e). The NTD and CTD are both composed of three α helices, and structurally resemble ubiquitin conjugating enzyme E2 K (PDB ID: 3K9O) and ubiquitin associated protein 1 (PDB ID: 4AE4), respectively, according to the Dali server^18^.

### Contribution of each domain of Regnase-1 to the mRNA binding activity

Although the PIN domain is responsible for the catalytic activity of Regnase-1^4^, the roles of the other domains are largely unknown. First, we evaluated a role of the NTD and ZF domains for mRNA binding by an *in vitro* gel shift assay (Fig. 1f). Fluorescently 5′-labeled RNA corresponding to nucleotides 82–106 of the IL-6 mRNA 3′UTR and the catalytically inactive mutant (D226N and D244N) of Regnase-1—hereafter referred to as the DDNN mutant—were utilized. Upon addition of a larger amount of Regnase-1, the fluorescence of free RNA decreased, indicating that Regnase-1 bound to the RNA. Based on the decrease in the free RNA fluorescence band, we evaluated the contribution of each domain of Regnase-1 to RNA binding. While the RNA binding ability was not significantly changed in the presence of NTD, it increased in the presence of the ZF domain (Fig. 1f,g and Supplementary Fig. 1). Direct binding of the ZF domain and RNA were confirmed by NMR spectral changes. The fitting of the titration curve of Y314 resulted in an apparent dissociation constant (*K*_*d*_) of 10 ± 1.1 μM (Supplementary Fig. 2). These results indicate that not only the PIN but also the ZF domain contribute to RNA binding, while the NTD is not likely to be involved in direct interaction with RNA.

### Contribution of each domain of Regnase-1 to RNase activity

In order to characterize the role of each domain in the RNase activity of Regnase-1, we performed an *in vitro* cleavage assay using fluorescently 5′-labeled RNA corresponding to nucleotides 82–106 of the IL-6 mRNA 3′UTR (Fig. 1g). Regnase-1 constructs consisting of NTD-PIN-ZF completely cleaved the target mRNA and generated the cleaved products. The apparent half-life (*T*_1/2_) of the RNase activity was about 20 minutes. Regnase-1 lacking the ZF domain generated a smaller but appreciable amount of cleaved product (*T*_1/2_ ~ 70 minutes), while those lacking the NTD did not generate cleaved products (*T*_1/2_ > 90 minutes). It should be noted that NTD-PIN(DDNN)-ZF, which possesses the NTD but lacks the catalytic residues in PIN, completely lost all RNase activity (Fig. 1g, right panel), as expected, confirming that the RNase catalytic center is located in the PIN domain. Taken together with the results in the previous section, we conclude that the NTD is crucial for the RNase activity of Regnase-1 *in vitro*, although it does not contribute to the direct mRNA binding.

### Dimer formation of the PIN domains

**Figure 2:**
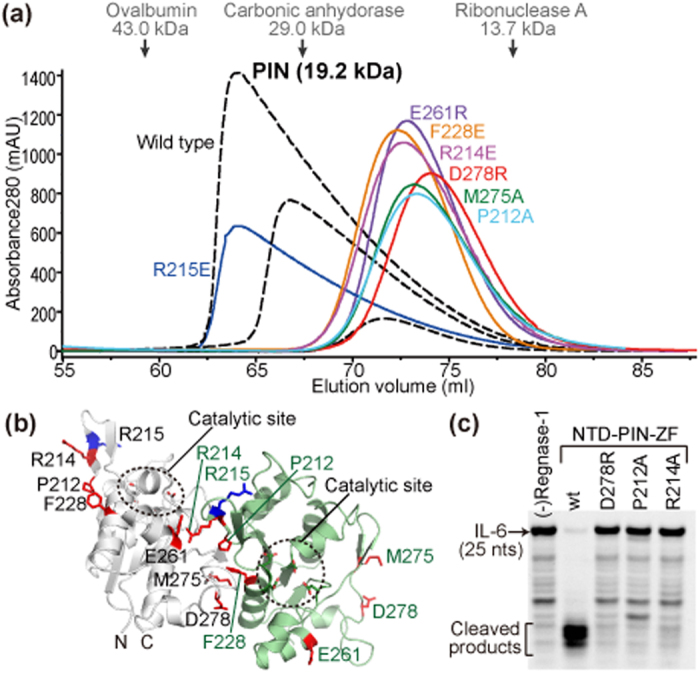
Head-to-tail oligomer formation of the PIN domain is crucial for the RNase activity of Regnase-1. (**a**) Gel filtration analyses of the PIN domain. Elution volumes of the standard marker proteins were indicated by arrows at the upper part. (**b**) Dimer structure of the PIN domain. Two PIN molecules in the crystal were colored white and green, respectively. Catalytic residues and mutated residues were shown in sticks. Residues important for the oligomeric interaction were colored red, while R215 that was dispensable for the oligomeric interaction was colored blue. (**c**) RNase activity of monomeric mutants for IL-6 mRNA was analyzed.

During purification by gel filtration, the PIN domain exhibited extremely asymmetric elution peaks in a concentration dependent manner (Fig. 2a). By comparison with the elution volume of standard marker proteins, the PIN domain was assumed to be in equilibrium between a monomer and a dimer in solution at concentrations in the 20–200 μM range. The crystal structure of the PIN domain has been determined in three distinct crystal forms with a space group of P3_1_21 (form I in this study and PDB ID 3V33), P3_2_21 (form II in this study), and P4_1_ (PDB ID 3V32 and 3V34), respectively^11^. We found that the PIN domain formed a head-to-tail oligomer that was commonly observed in all three crystal forms in spite of the different crystallization conditions (Supplementary Fig. 3). Mutation of Arg215, whose side chain faces to the opposite side of the oligomeric surface, to Glu preserved the monomer/dimer equilibrium, similar to the wild type. On the other hand, single mutations of side chains involved in the PIN–PIN oligomeric interaction resulted in monomer formation, judging from gel filtration (Fig. 2a,b). Wild type and monomeric PIN mutants (P212A and D278R) were also analyzed by NMR. The spectra indicate that the dimer interface of the wild type PIN domain were significantly broadened compared to the monomeric mutants (Supplementary Fig. 4). These results indicate that the PIN domain forms a head-to-tail oligomer in solution similar to the crystal structure. Interestingly, the monomeric PIN mutants P212A, R214A, and D278R had no significant RNase activity for IL-6 mRNA *in vitro* (Fig. 2c). The side chains of these residues point away from the catalytic center on the same molecule (Fig. 2b). Therefore, we concluded that head-to-tail PIN dimerization, together with the NTD, are required for Regnase-1 RNase activity *in vitro*.

### Domain-domain interaction between the NTD and the PIN domain

**Figure 3:**
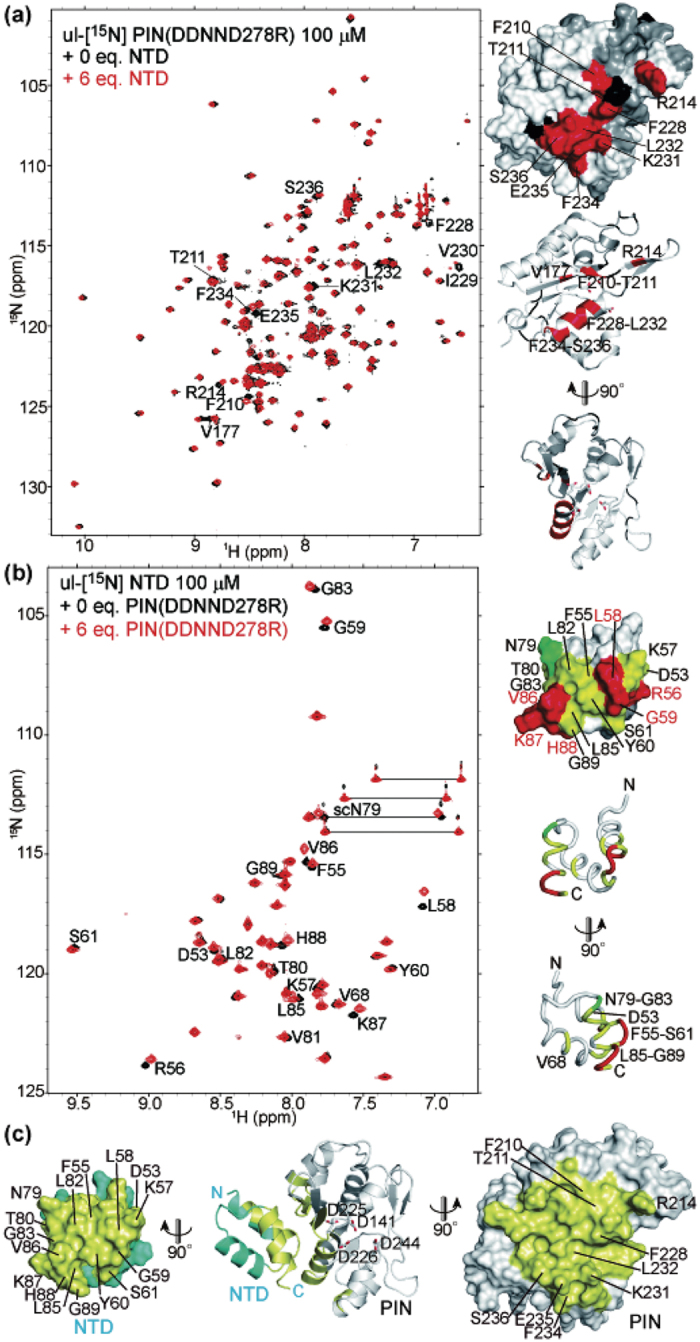
Domain-domain interaction between the NTD and the PIN domain. (**a**) NMR analyses of the NTD-binding to the PIN domain. The residues with significant chemical shift changes were labeled in the overlaid spectra (left) and colored red on the surface and ribbon structure of the PIN domain (right). Pro and the residues without analysis were colored black and gray, respectively. (**b**) NMR analyses of the PIN-binding to the NTD. The residues with significant chemical shift changes were labeled in the overlaid spectra (left) and colored red, yellow, or green on the surface and ribbon structure of the NTD. S62 was colored gray and excluded from the analysis, due to low signal intensity. (**c**) Docking model of the NTD and the PIN domain. The NTD and the PIN domain are shown in cyan and white, respectively. Residues in close proximity (<5 Å) to each other in the docking structure were colored yellow. Catalytic residues of the PIN domain are shown in sticks, and the residues that exhibited significant chemical shift changes in (**a,b**) were labeled.

While the NTD does not contribute to RNA binding (Fig. 1f,g, and Supplementary Fig. 1), it increases the RNase activity of Regnase-1 (Fig. 1h). In order to gain insight into the molecular mechanism of the NTD-mediated enhancement of Regnase-1 RNase activity, we further investigated the domain-domain interaction between the NTD and the PIN domain using NMR. We used the catalytically inactive monomeric PIN mutant possessing both the DDNN and D278R mutations to avoid dimer formation of the PIN domain. The NMR signals from the PIN domain (residues V177, F210-T211, R214, F228-L232, and F234-S236) exhibited significant chemical shift changes upon addition of the NTD (Fig. 3a). Likewise, upon addition of the PIN domain, NMR signals derived from R56, L58-G59, and V86-H88 in the NTD exhibited large chemical shift changes and residues D53, F55, K57, Y60-S61, V68, T80-G83, L85, and G89 of the NTD as well as side chain amide signals of N79 exhibited small but appreciable chemical shift changes (Fig. 3b and Supplementary Fig. 5). These results clearly indicate a direct interaction between the PIN domain and the NTD. Based on the titration curve for the chemical shift changes of L58, the apparent *K*_*d*_ between the isolated NTD and PIN was estimated to be 110 ± 5.8 μM. Considering the fact that the NTD and PIN domains are attached by a linker, the actual binding affinity is expected much higher in the native protein. Mapping the residues with chemical shift changes reveals the putative PIN/NTD interface, which includes a helix that harbors catalytic residues D225 and D226 on the PIN domain (Fig. 3a). Interestingly, the putative binding site for the NTD overlaps with the PIN-PIN dimer interface, implying that NTD binding can “terminate” PIN-PIN oligomerization (Fig. 2b). An *in silico* docking of the NTD and PIN domains using chemical shift restraints provided a model consistent with the NMR experiments (Fig. 3c).

### Residues critical for Regnase-1 RNase activity

**Figure 4:**
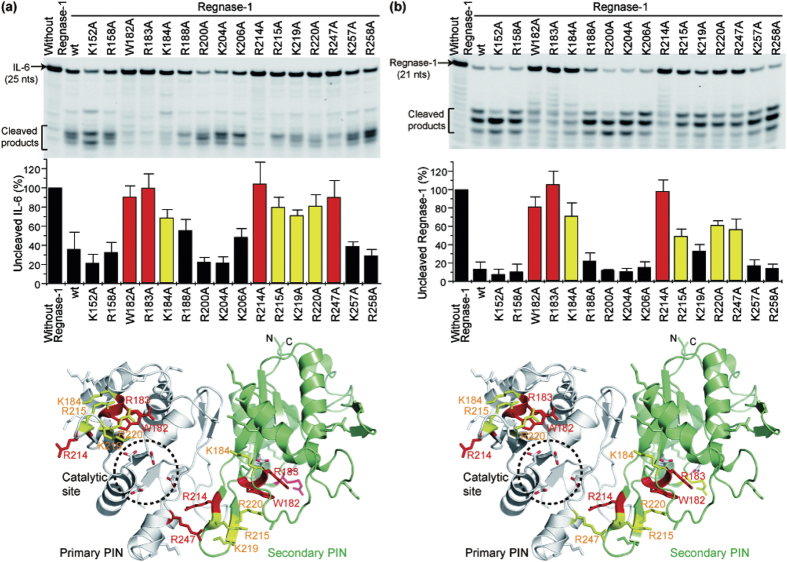
Critical residues in the PIN domain for the RNase activity of Regnase-1. (**a**) *In vitro* cleavage assay of basic residue mutants for IL-6 mRNA. The results indicate mean ± SD of four independent experiments. **(b)** *In vitro* cleavage assay of basic residue mutants for Regnase-1 mRNA. The results indicate mean ± SD of three independent experiments. The fluorescence intensity of the uncleaved mRNA was quantified and the results were mapped on the PIN dimer structure. Mutated basic residues were shown in sticks and those with significantly reduced RNase activities were colored red or yellow.

To gain insight into the residues critical for Regnase-1 RNase activity, each basic or aromatic residue located around the catalytic site of the PIN oligomer was mutated to alanine, and the oligomerization and RNase activity were investigated (Fig. 4). From the gel filtration assays, all mutants except R214A formed dimers, suggesting that any lack of RNase activity in the mutants, except R214A, was directly due to mutational effects of the specific residues and not to abrogation of dimer formation. The W182A, R183A, and R214A mutants markedly lost cleavage activity for IL-6 mRNA as well as for Regnase-1 mRNA. The K184A, R215A, and R220A mutants moderately but significantly decreased the cleavage activity for both target mRNAs. The importance of K219 and R247 was slightly different for IL-6 and Regnase-1 mRNA; both K219 and R247 were more important in the cleavage of IL-6 mRNA than for Regnase-1 mRNA. The other mutated residues—K152, R158, R188, R200, K204, K206, K257, and R258—were not critical for RNase activity. The importance of residues W182 and R183 can readily be understood in terms of the monomeric PIN structure as they are located near to the RNase catalytic site; however, the importance of residue K184, which points away from the active site is more easily rationalized in terms of the oligomeric structure, in which the “secondary” chain’s residue K184 is positioned near the “primary” chain’s catalytic site (Fig. 4). In contrast, R214 is important for oligomerization of the PIN domain and the “secondary” chain’s residue R214 is also positioned near the “primary” chain’s active site within the dimer interface. It should be noted that the putative-RNA binding residues K184 and R214 are unique to Regnase-1 among PIN domains.

### Molecular mechanism of target mRNA cleavage by the PIN dimer

**Figure 5:**
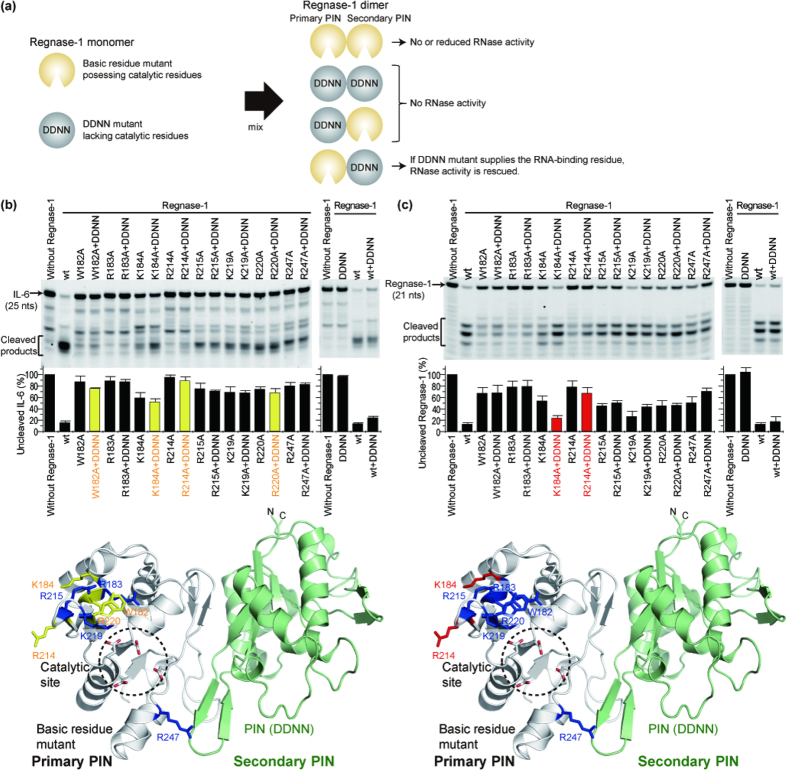
Heterodimer formation by combination of the Regnase-1 basic residue mutants and the DDNN mutant restored the RNase activity. (**a**) Cartoon representation of the concept of the experiment. (**b**) *In vitro* cleavage assay of Regnase-1 for IL-6 mRNA. (**c**) *In vitro* cleavage assay of Regnase-1 for Regnase-1 mRNA. The results indicate mean ± SD of three independent experiments. The fluorescence intensity of the uncleaved mRNA was quantified and the results were mapped on the PIN dimer. The mutations whose RNase activities were not increased in the presence of DDNN mutant were colored in blue on the primary PIN. The mutations whose RNase activities were restored in the presence of DDNN mutant were colored in red or yellow on the primary PIN.

Our mutational experiments indicated that the observed dimer is functional and that the role of the secondary PIN domain is to position Regnase-1-unique RNA binding residues near the active site of the primary PIN domain. If this model is correct, then we reasoned that a catalytically inactive PIN and a PIN lacking the putative RNA-binding residues ought to be inactive in isolation but become active when mixed together. In order to test this hypothesis, we performed *in vitro* cleavage assays using combinations of Regnase-1 mutants that had no or decreased RNase activities by themselves (Fig. 5). One group consisted of catalytically active PIN domains with mutation of basic residues found in the previous section to confer decreased RNase activity (Fig. 4). These were paired with a DDNN mutant that had no RNase activity by itself. When any members of the two groups are mixed, two kinds of heterodimers can be formed: one is composed of a DDNN primary PIN and a basic residue mutant secondary PIN and is expected to exhibit no RNase activity; the other is composed of a basic residue mutant primary PIN and a DDNN secondary PIN and is predicted to rescue RNase activity (Fig. 5a). When we compared the fluorescence intensity of uncleaved IL-6 mRNA, basic residue mutants W182A, K184A, R214A, and R220A were rescued upon addition of the DDNN mutant (Fig. 5b). Consistently, when we compared the fluorescence intensity of the uncleaved Regnase-1 mRNA, basic residue mutants K184A and R214A were rescued upon addition of the DDNN mutant (Fig. 5c). Rescue of K184A and R214A by the DDNN mutant was also confirmed by a significant increase in the cleaved products. This is particularly significant because the side chains of K184 and R214 in the primary PIN are oriented away from their own catalytic center, while those in the secondary PIN face toward the catalytic center of the primary PIN. R214 is an important residue for dimer formation as shown in Fig. 2, therefore, R214A in the secondary PIN cannot dimerize. According to the proposed model, an R214A PIN domain can only form a dimer when the DDNN PIN acts as the secondary PIN. Taken together, the rescue experiments above support the proposed model in which the head-to-tail dimer is functional *in vitro*.

## Discussion

We determined the individual domain structures of Regnase-1 by NMR and X-ray crystallography. Although the function of the CTD remains elusive, we revealed the functions of the NTD, PIN, and ZF domains. A Regnase-1 construct consisting of PIN and ZF domains derived from *Mus musculus* was crystallized; however, the electron density of the ZF domain was low, indicating that the ZF domain is highly mobile in the absence of target mRNA or possibly other protein-protein interactions. Our NMR experiments confirmed direct binding of the ZF domain to IL-6 mRNA with a *K*_*d*_ of 10 ± 1.1 μM. Furthermore, an *in vitro* gel shift assay indicated that Regnase-1 containing the ZF domain enhanced target mRNA-binding, but the protein-RNA complex remained in the bottom of the well without entering into the polyacrylamide gel. These results indicate that Regnase-1 directly binds to RNA and precipitates under such experimental conditions. Due to this limitation, it is difficult to perform further structural analyses of mRNA-Regnase-1 complexes by X-ray crystallography or NMR.

The previously reported crystal structure of the Regnase-1 PIN domain derived from *Homo sapiens* is nearly identical to the one derived from *Mus musculus* in this study, with a backbone RMSD of 0.2 Å. The amino acid sequences corresponding to PIN (residues 134–295) are the two non-identical residues are substituted with similar amino acids. Both the mouse and human PIN domains form head-to-tail oligomers in three distinct crystal forms. Rao and co-workers previously argued that PIN dimerization is likely to be a crystallographic artifact with no physiological significance, since monomers were dominant in their analytical ultra-centrifugation experiments^11^. In contrast, our gel filtration data, mutational analyses, and NMR spectra all indicate that the PIN domain forms a head-to-tail dimer in solution in a manner similar to the crystal structure. This inconsistency might be due to difference in the analytical methods and/or protein concentrations used in each experiment, since the oligomer formation of PIN was dependent on the protein concentration in our study.

Single mutations to residues involved in the putative oligomeric interaction of PIN monomerized as expected and these mutants lost their RNase activity as well. Since the NMR spectra of monomeric mutants overlaps with those of the oligomeric forms, it is unlikely that the tertiary structure of the monomeric mutants were affected by the mutations. (Supplementary Fig. 4b,c). Based on these observations, we concluded that PIN-PIN dimer formation is critical for Regnase-1 RNase activity *in vitro*. Within the crystal structure of the PIN dimer, the Regnase-1 specific basic regions in both the “primary” and “secondary” PINs are located around the catalytic site of the primary PIN (Supplementary Fig. 6). Moreover, our structure-based mutational analyses showed these two Regnase-1 specific basic regions were essential for target mRNA cleavage *in vitro*.

The cleavage assay also showed that the NTD is crucial for efficient mRNA cleavage. Moreover, we found that the NTD associates with the oligomeric surface of the primary PIN, docking to a helix that harbors its catalytic residues (Figs 2b and 3a). Taken together, this suggests that the NTD and the PIN domain compete for a common binding site. The affinity of the domain-domain interaction between two PIN domains (*K*_*d*_ = ~10^−4^ M) is similar to that of the NTD-PIN (*K*_*d*_ = 110 ± 5.8 μM) interactions; however, the covalent connection corresponding to residues 90–133 between the NTD and the primary PIN will greatly enhance the intramolecular domain interaction in the case of full-length Regnase-1. While further analyses are necessary to prove this point, our preliminary docking and molecular dynamics simulations indicate that NTD-binding rearranges the catalytic residues of the PIN domain toward an active conformation suitable for binding Mg^2+^. In this context, it is interesting that, in response to TCR stimulation, Malt1 cleaves Regnase-1 at R111 to control immune responses *in vivo*^19^. This result is consistent with a model in which the NTD acts as an enhancer, and cleavage of the linker lowers enzymatic activity dramatically.

Based on these structural and functional analyses of Regnase-1 domain-domain interactions, we performed docking simulations of the NTD, PIN dimer, and IL-6 mRNA. We incorporated information from the cleavage site of IL-6 mRNA *in vitro* is indicated by denaturing polyacrylamide gel electrophoresis (Supplementary Fig. 7a,b). The docking result revealed multiple RNA binding modes that satisfied the experimental results *in vitro* (Supplementary Fig. 7c,d), however, it should be noted that, *in vivo*, there would likely be many other RNA-binding proteins that would protect loop regions from cleavage by Regnase-1.

**Figure 6:**
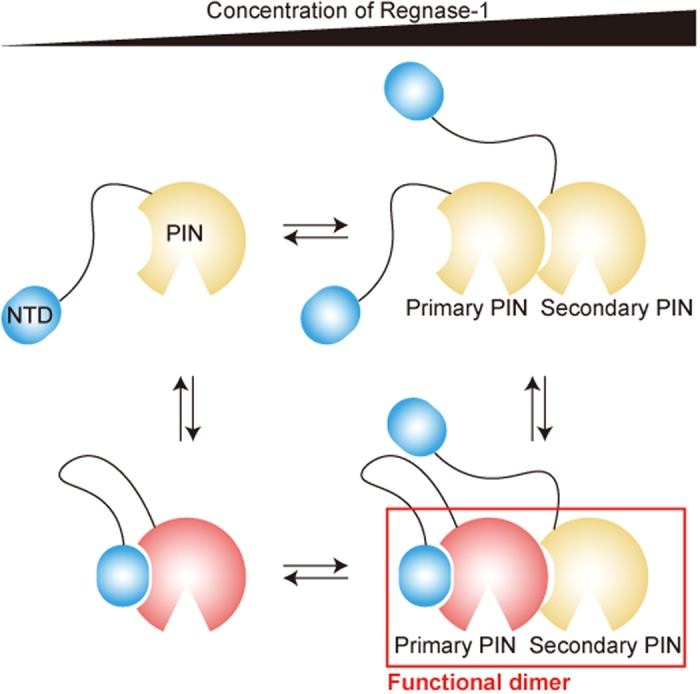
Schematic representation of regulation of the Regnase-1 catalytic activity through the domain-domain interactions.

The overall model of regulation of Regnase-1 RNase activity through domain-domain interactions *in vitro* is summarized in Fig. 6. In the absence of target mRNA, the PIN domain forms head-to-tail oligomers at high concentration. A fully active catalytic center can be formed only when the NTD associates with the oligomer surface of the PIN domain, which terminates the head-to-tail oligomer formation in one direction (primary PIN), and forms a functional dimer together with the neighboring PIN (secondary PIN). While further investigations on the domain-domain interactions of Regnase-1 *in vivo* are necessary, these intramolecular and intermolecular domain interactions of Regnase-1 appear to structurally constrain Regnase-1activity, which, in turn, enables tight regulation of immune responses.

## Methods

### Protein expression and purification

The DNA fragment encoding Regnase-1 derived from *Mus musculus* was cloned into pGEX6p vector (GE Healthcare). All the mutants were generated by PCR-mediated site-directed mutagenesis and confirmed by the DNA sequence analyses. As a catalytically deficient mutant, both Asp226 and Asp244 at the catalytic center of PIN were mutated to Asn, which is referred to as DDNN mutant. Regnase-1 was expressed at 16 °C using the *Escherichia coli* Rosetta^TM^(DE3)pLysS strain. After purification with a GST-affinity resin, an N-terminal GST tag was digested by HRV-3 C protease. NTD was further purified by gel filtration chromatography using a HiLoad 16/60 Superdex 75 pg (GE Healthcare). The other domains were further purified by cation exchange chromatography using Resource S (GE Healthcare), followed by gel filtration chromatography using a HiLoad 16/60 Superdex 75 pg (GE Healthcare). Uniformly ^15^N or ^13^C, ^15^N-double labeled proteins for NMR experiments were prepared by growing *E. coli* host in M9 minimal medium containing ^15^NH_4_Cl, unlabeled glucose and ^15^N CELTONE^®^ Base Powder (CIL) or ^15^NH_4_Cl, ^13^C_6_-glucose, and^13^C, ^15^N CELTONE^®^ Base Powder (CIL), respectively.

### X-ray crystallography

Crystallization was performed using the sitting drop vapor diffusion method at 20 °C and two crystal forms (I and II) were obtained. In the case of form I crystals, drops (0.5 μl) of 6 mg/ml selenomethionine-labeled Regnase-1 PIN-ZF (residues 134–339 derived from *Mus musculus*) in 20 mM HEPES-NaOH (pH 6.8), 200 mM NaCl and 5 mM DTT were mixed with reservoir solution consisting of 1 M (NH_4_)_2_HPO_4_, 200 mM NaCl and 100 mM sodium citrate (pH 5.5) whereas in the case of form II crystals, drops (0.5 μl) of 6 mg/ml native Regnase-1 PIN-ZF (residues 134–339) in 20 mM HEPES-NaOH (pH 6.8), 200 mM NaCl and 5 mM DTT were mixed with reservoir solution consisting of 1.7 M NaCl and 100 mM HEPES-NaOH (pH 7.0). Diffraction data were collected at a Photon Factory Advanced Ring beamline NE3A (form I) or at a SPring-8 beamline BL41XU (form II), and were processed with HKL2000^20^. The structure of the form I crystal was determined by the multiple anomalous dispersion (MAD) method. Nine Se sites were found using the program SOLVE^21^; however, the electron density obtained by MAD phases calculated using SOLVE was not good enough to build a model even after density modification using the program RESOLVE^22^. Then the program CNS^23^ was used to find additional three Se sites and calculate MAD phases using 12 Se sites. The electron density after density modification using CNS was good enough to build a model. Structure of the form II crystal was determined by the molecular replacement method using CNS and using the structure of the form I crystal as a search model. For all structures, further model building was performed manually with COOT^24^, and TLS and restrained refinement using isotropic individual B factors was performed with REFMAC5^25^ in the CCP4 program suite^26^. Crystallographic parameters are summarized in Supplementary Table 1.

### NMR measurements

All NMR experiments were carried out at 298 K on Inova 500-MHz, 600-MHz, and 800-MHz spectrometer (Agilent). The NMR data were processed using the NMRPipe^27^, the Olivia (fermi.pharm.hokudai.ac.jp/olivia/), and the Sparky program (Sparky3, University of California, San Francisco).

For structure calculation, NOE distance restraints were obtained from 3D ^15^N-NOESY-HSQC (100 ms mixing time for the NTD, 75 ms mixing time for the ZF domain and the CTD) and ^13^C-NOESY-HSQC spectra (100 ms mixing time for the NTD, 75 ms mixing time for the ZF domain and the CTD). The NMR structures were determined using the CANDID/CYANA2.1^28^. Dihedral restraints were derived from backbone chemical shifts using TALOS^29^.

For the domain-domain interaction analyses between the NTD and the PIN domain, ^1^H-^15^N HSQC spectra of uniformly ^15^N-labeled proteins in the concentration of 100 μM were obtained in the presence of 3 or 6 molar equivalents of unlabeled proteins.

### Preparation of RNAs

The fluorescently labeled RNAs at the 5′-end by 6-FAM were purchased from SIGMA-ALDORICH. The RNA sequences used in this study were shown below.

IL-6 mRNA 3′UTR (82–106): 5′-UGUUGUUCUCUACGAAGAACUGACA-3′ (25 nts)

Regnase-1 mRNA 3′UTR (191–211): 5′- CUGUUGAUACACAUUGUAUCU-3′ (21 nts)

### Electrophoretic mobility shift assay

Catalytically deficient Regnase-1 proteins, containing DDNN mutations, and 5′-terminally 6-FAM labeled RNAs were incubated in the RNA-binding buffer (20 mM HEPES-NaOH (pH 6.8), 150 mM NaCl, 1 mM DTT, 10% glycerol (v/v), and 0.1% NP-40 (v/v)) at 4 °C for 30 minutes, then analyzed by non-denaturing polyacrylamide gel electrophoresis. The electrophoreses were performed at 4 °C using the 7.5% polyacrylamide (w/v) gel (monomer : bis = 29 : 1) in the electrophoresis buffer (25 mM Tris-HCl (pH 7.5) and 200 mM glycine). The fluorescence of 6-FAM labeled RNA was directly detected at the excitation wavelength of 460 nm with a fluorescence filter (Y515-Di) using a fluoroimaging analyzer (LAS-4000 (FUJIFILM)). The fluorescence intensity of each sample was quantified using ImageJ software.

### *In vitro* RNA cleavage assay

Regnase-1 (2 μM) and 5′-terminally 6-FAM labeled RNA (1 μM) were incubated in the RNA-cleavage buffer (20 mM Tris-HCl (pH 7.5), 150 mM NaCl, 5 mM MgCl_2_, and 1 mM DTT) at 37 °C. For the assay using combinations of Regnase-1 mutants, equimolar amounts of Regnase-1 mutants (2 μM each) were mixed with fluorescently labeled RNA (1 μM). After incubation for 30–120 minutes, the reaction was stopped by the addition of 1.5-fold volume of denaturing buffer containing 8 M urea and 100 mM EDTA, and samples were boiled. The electrophoreses were performed at room temperature using the 8 M urea containing denaturing gel with 20% polyacrylamide (w/v) (monomer : bis = 19 : 1) in 0.5 × TBE as the electrophoresis buffer.

### Docking calculations

For docking NTD to PIN, OSCAR-star^30^ was first used to rebuild sidechains in the head-to-tail PIN dimer. Docking was carried out by surFit (http://sysimm.ifrec.osaka-u.ac.jp/docking/main/) with restraints obtained from NMR data (Fig. 3a,b) as follows. NTD: R56, L58, G59, V86, K87, H88; PIN: V177, F210, T211, R214, F228, I229, V230, K231, L232, F234, D235, S236. Top-scoring model was selected.

For docking IL-6 mRNA 3′UTR to the PIN dimer, each domain of the PIN dimer structure was superimposed onto the PIN dimer of the human X-ray structure (PDB ID: 3V34) in order to graft both water molecules and Mg^2+^ ions to the mouse model. Each IL-6 representative structure was submitted to the HADDOCK 2.0 server, for total of 10 independent jobs. In order to be consistent with the cleavage assay, active residues consisted of all nucleotides in RNA, Mg^2+^ and W182, R183, K184, R188, R214, R215, K219, R220, and R247 in the protein. Docked models were selected based on the following criteria: one heavy atom within 7, 8, or 9^th^ nucleotide from the 5′ end was <5 Å from the Mg^2+^ ion on the primary PIN. Further classification was done manually in order to divide the selected models into two clusters.

## Additional Information

**Accession codes:** The crystal structure of the Regnase-1 PIN domain has been deposited in the Protein Data Bank (accession codes: 5H9V (Form I) and 5H9W (Form II)). The chemical shift assignments of the NTD, the ZF domain, and the CTD have been deposited at Biological Magnetic Resonance Bank (accession codes: 25718, 25719, and 25720, respectively), and the coordinates for the ensemble have been deposited in the Protein Data Bank (accession codes: 2N5J, 2N5K, and 2N5L, respectively). 

**How to cite this article**: Yokogawa, M. *et al.* Structural basis for the regulation of enzymatic activity of Regnase-1 by domain-domain interactions. *Sci. Rep.* **6**, 22324; doi: 10.1038/srep22324 (2016).

## Supplementary Material

Supplementary Information
