# Supplementary material for: Structural basis for the regulation of enzymatic activity of Regnase-1 by domain-domain interactions
Source: Sci Rep. 2016 Mar 1;6:22324. doi: 10.1038/srep22324 (PMC4772114; doi:10.1038/srep22324)
Supplement: Supplementary Information [file srep22324-s1.pdf]

Structural basis for the regulation of enzymatic activity of Regnase-1 by domain-domain interactions

Mariko Yokogawa[1], Takashi Tsushima[2], Nobuo N. Noda[3], Hiroyuki Kumeta[1], Yoshiaki Enokizono[1], Kazuo Yamashita[4], Daron M. Standley[4, 6], Osamu Takeuchi[4, 5, 6], Shizuo Akira[4, 5], and Fuyuhiko Inagaki[1,*]

Supplementary Information

Supplementary Tables 1-2

Supplementary Figures 1-7

Supplementary Data

Supplementary Table 1 Data collection, phasing and refinement statistics for the PIN domain

	Form I				Form II
Data collection statistics	Peak	Edge	High Remote		
Wavelength (Å)	0.97899	0.97928	0.96405	0.97899	1.00000
Space group		$P3_221$		$P3_221$	$P3_121$
Cell parameters (Å)		a = 113.36		a = 113.37	a = 113.33
Resolution range (Å)	47.44–3.00	47.44–3.00	47.36–3.00	47.49-2.75	26.94-2.60
Observed reflections	429,716	408,689	354,889	263,916	176,336
Unique reflections	52,901	53,228	51,115	33,467	16,940
Completeness (%)	98.4 (88.3)	99.4 (95.3)	97.2 (82.6)	90.5 (54.1)	92.9 (67.8)
R_{sym}	0.073 (0.304)	0.065 (0.257)	0.077 (0.330)	0.103 (0.514)	0.067 (0.255)
$\langle I/\sigma(I) \rangle$	37.0 (13.8)	59.9 (16.8)	49.4 (12.6)	22.2 (3.1)	34.3 (7.9)
Phasing statistics					
Resolution range (Å)		47.5-3.3			
No. of Se sites		12			
Mean figure of merit		0.708			
Refinement statistics					
Resolution range (Å)				47.53-2.75	26.95-2.60
No. of protein atoms				5,285	2,658
No. of sodium ions				4	2
No. of water molecules					34
R / R_{free}				0.191/0.226	0.194/0.231
rmsd from ideality					
bond length (Å)				0.014	0.015
angles (º)				1.7	1.7

Values in parentheses refer to the outer shell.

Supplementary Table 2 NMR and refinement statistics for NTD, ZF and CTD

	**NTD**	**ZF**	**CTD**
**NMR distance and dihedral constraints**			
Distance constraints			
Total NOE	950	350	1116
Intra-residue	251	85	342
Inter-residue	699	265	774
Sequential (|i-j| = 1)	268	108	305
Medium-range (|i-j| < 4)	284	101	290
Long-range (|i-j > 5)	147	56	179
Intermolecular	0	0	0
Hydrogen bonds	8	0	0
Total dihedral angle restraints	70	18	78
phi	**35**	**9**	**39**
psi	**35**	**9**	**39**
**Structure statistics**			
Violations (mean and s.d.)			
Distance constraints (Å)	0.0033±0.0013	0.0095±0.0034	0.0084±0.0022
Dihedral angle constraints (°)	0.051±0.026	0.48±0.30	0.48±0.20
Max. dihedral angle violation (°)	0.17	2.47	2.63
Max. distance constraint violation (Å)	0.06	0.18	0.08
Deviations from idealized geometry			
Bond lengths (Å)			
Bond angles (°)			
Impropers (°)			
Average pairwise r.m.s.d.** (Å)			
Heavy	0.80	1.35	0.94
Backbone	0.34	0.49	0.31

** Pairwise r.m.s.d. was calculated among 20 refined structures, ranges 45-88 for NTD, ranges 301-326 for ZF and ranges 544-593 for CTD.

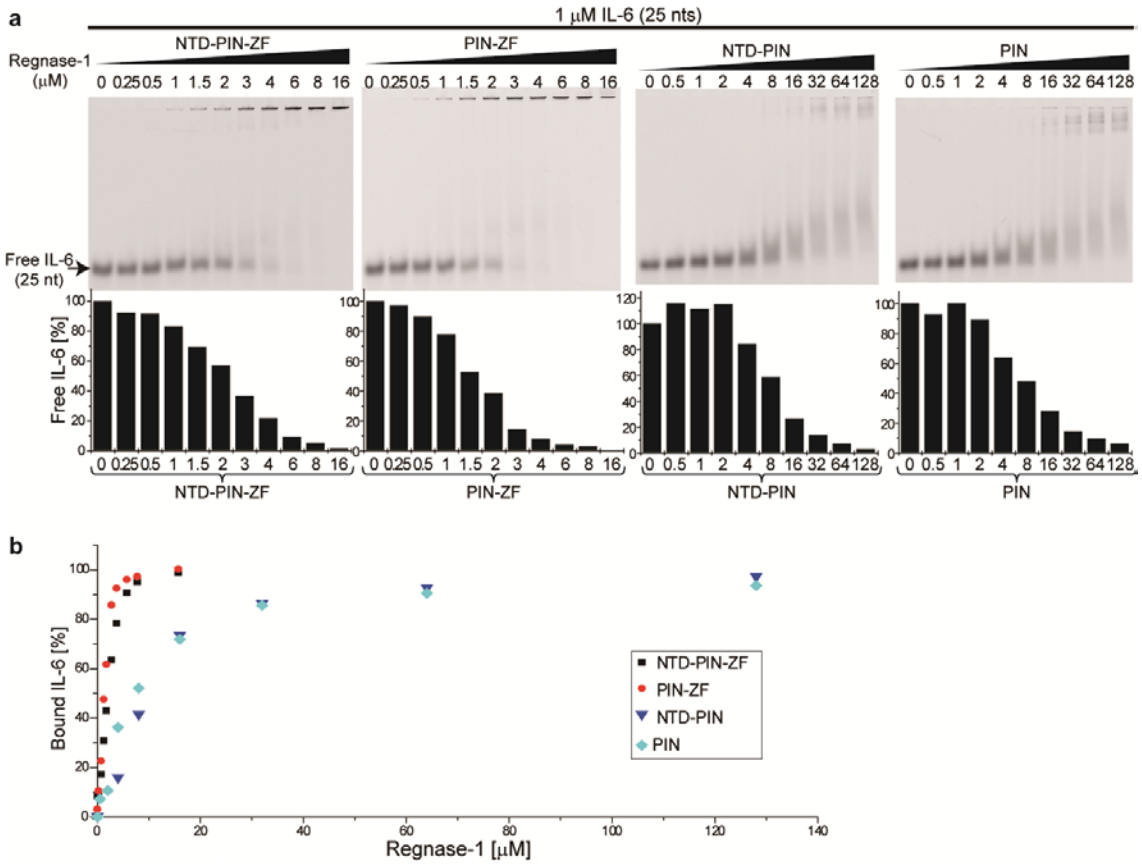

Supplementary Figure 1 Apparent affinity of Regnase-1 and IL-6 mRNA analyzed by *in vitro* gel shift assay. (a) Fluorescently labeled IL-6 mRNA was mixed with various amount of Regnase-1 and analyzed by non-denaturing polyacrylamide gels. The fluorescence intensity of free IL-6 mRNA in each sample was quantified. **(b)** Binding of Regnase-1 and IL-6 mRNA was plotted. The percentage of the bound IL-6 was calculated based on the fluorescence intensities of the free IL-6 quantified in (a).

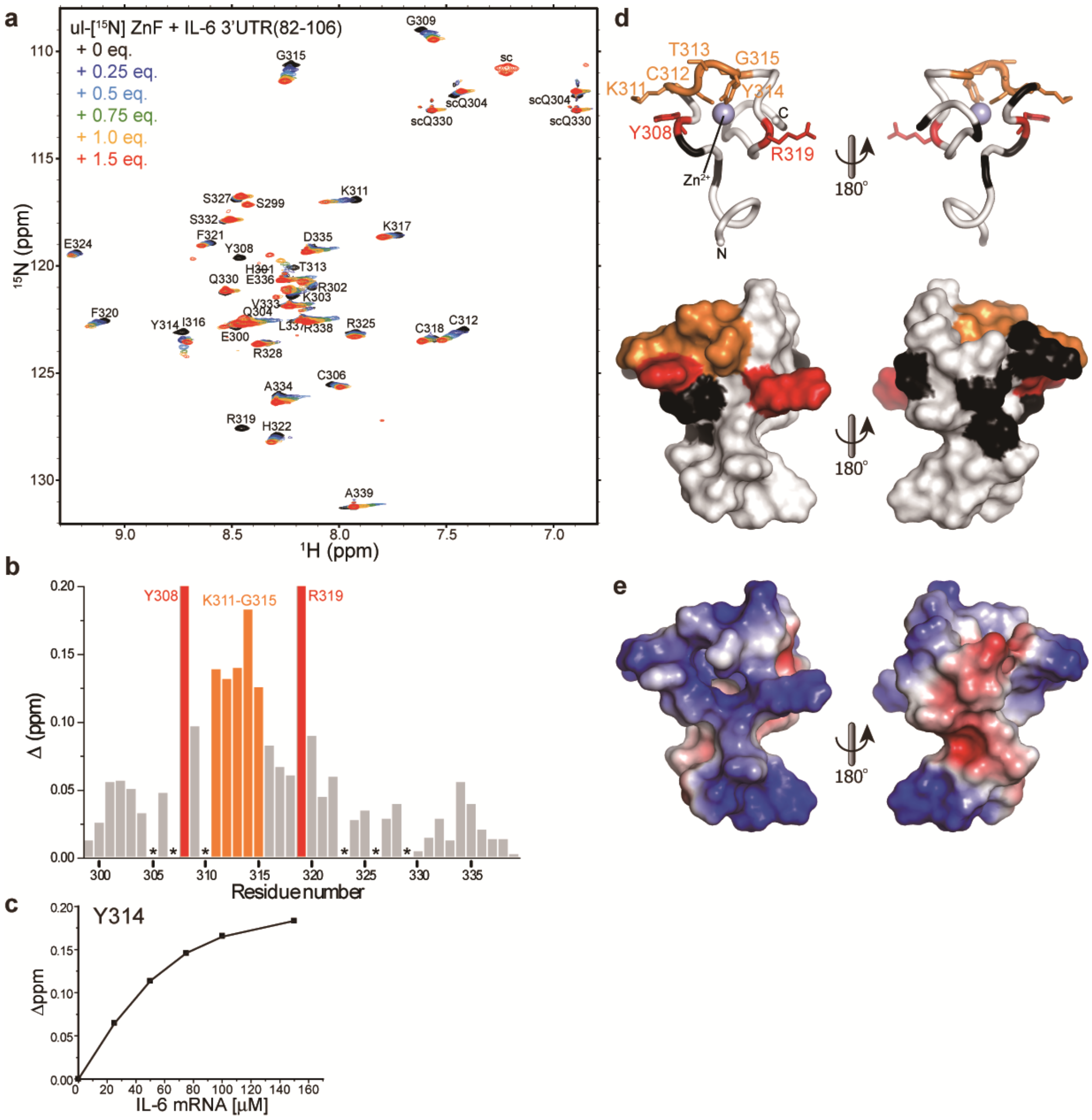

Supplementary Figure 2 NMR analyses of the interaction between the ZF domain and IL-6 mRNA. (a) ^{1}H-^{15}N HSQC spectra of uniformly ^{15}N-labeled ZF domain (residues 299-339, at the concentration of 100 μM) in the presence of 0, 0.25, 0.5, 0.75, 1.0, and 1.5 molar equivalents of IL-6 mRNA were overlaid in black, blue, cyan, green, yellow, and red, respectively. The unlabeled RNA corresponding to nucleotides 82-106 of the IL-6 mRNA 3'UTR was purchased from Hokkaido System Science Co. **(b)** The chemical shift difference of

each residue upon addition of 1.5 molar equivalents of IL-6 mRNA was calculated using the following equation: $\Delta(\text{ppm}) = ((\Delta\delta H_N)^2 + (\Delta\delta N/6.5)^2)^{0.5}$, where $\Delta\delta H_N$ and $\Delta\delta N$ are the changes in the chemical shifts along the ^1H and ^{15}N axes, respectively, and plotted against residue numbers of ZF. The most significant spectral change was detected for the signals of Y308 and R319 that disappeared upon addition of 0.25 molar equivalent of IL-6 mRNA, therefore Δ(ppm) of these signals were plotted as 0.2 and colored in red. The residues with $0.1 < \Delta(\text{ppm}) < 0.2$ were colored in orange. Pro or the residues without analysis were shown with asterisks. **(c)** The K_d value was estimated by a nonlinear least squares analysis using Origin5.0 (OriginLab) and the equation $\Delta\text{ppm} = \Delta\text{ppm}_{max} \times ([\text{IL-6 mRNA}]_{tot} + [\text{ZF}]_{tot} + K_d - (([\text{IL-6 mRNA}]_{tot} + [\text{ZF}]_{tot} + K_d)^2 - 4 \times [\text{IL-6 mRNA}]_{tot} \times [\text{ZF}]_{tot})^{0.5}) / (2 \times [\text{ZF}]_{tot})$, where Δppm is the observed chemical shift change at the given total IL-6 mRNA concentration, Δppm_{max} is the chemical shift change at saturation, and $[\text{ZF}]_{tot}$ and $[\text{IL-6 RNA}]_{tot}$ are the total concentrations of ZF and IL-6 RNA, respectively. **(d)** The result was mapped on the structure of ZF. Pro or the residues without analysis were colored in black. **(d)** Electric potential of ZF were exhibited.

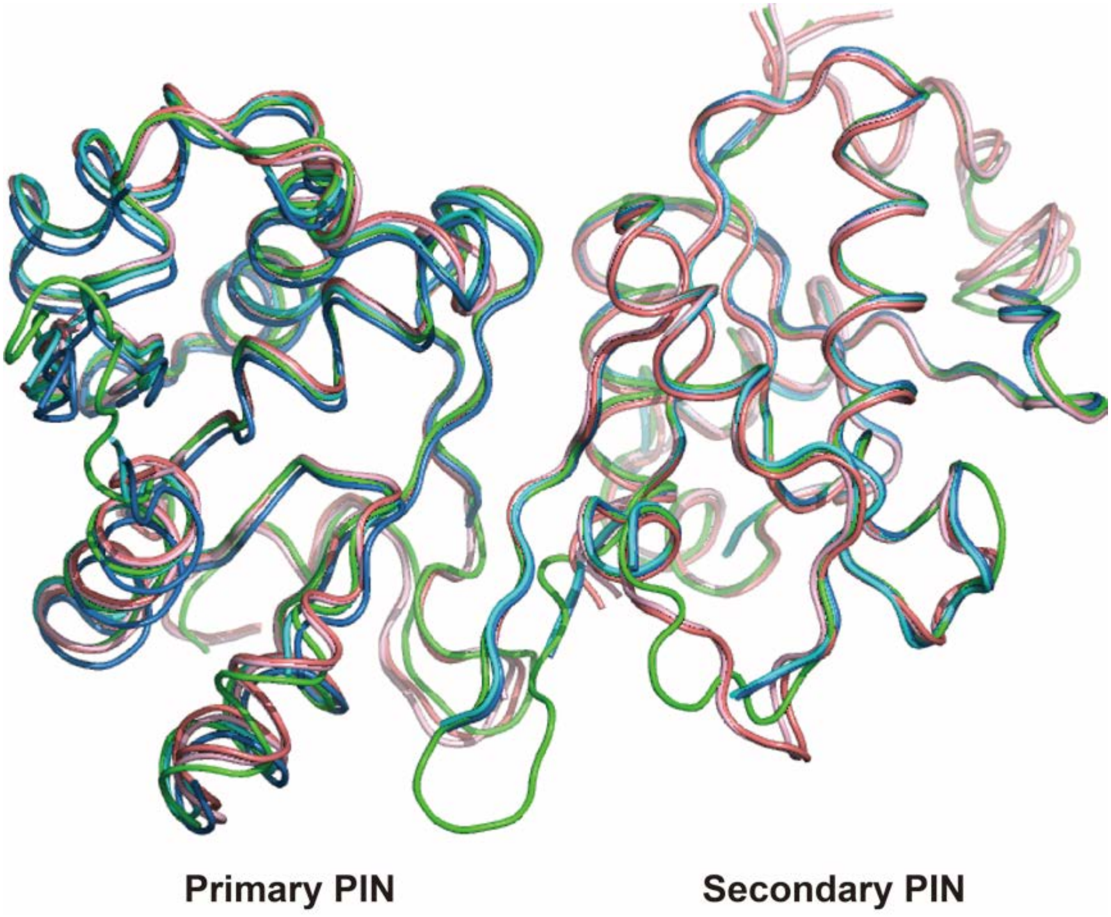

Supplementary Figure 3 Head-to-tail interactions observed in three distinct crystal forms. Form I structure was superimposed with the structures in form II and 3V32 using the program COOT (5). Form I was shown green, two molecules in Form II were shown blue and cyan, and two molecules in 3V32 were shown pink and red, respectively.

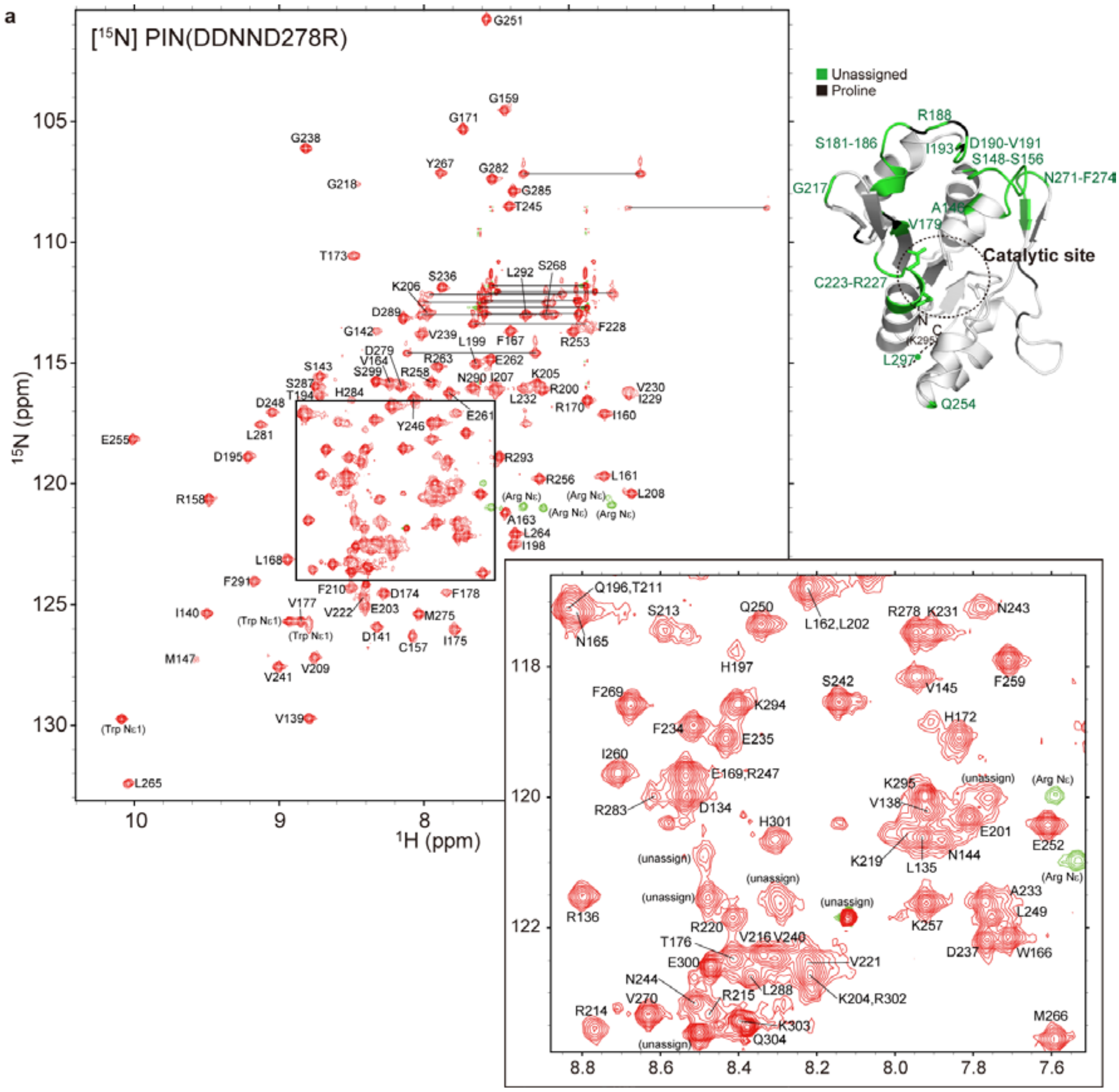
a
[15N] PIN(DDNND278R)
15N (ppm)
1H (ppm)
Unassigned
Proline
Catalytic site

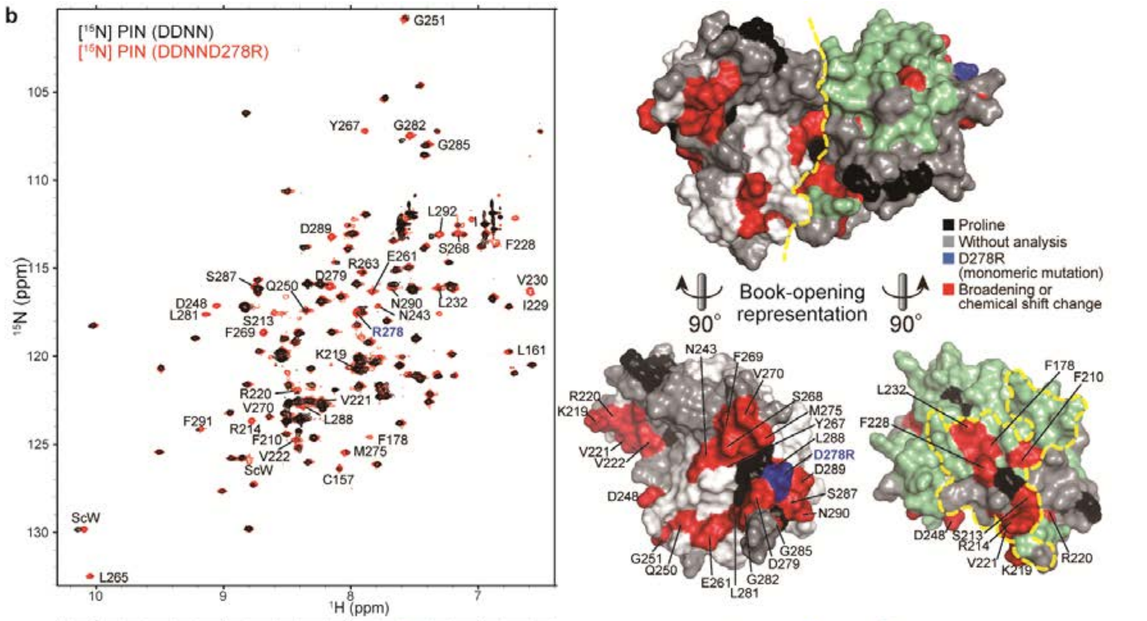

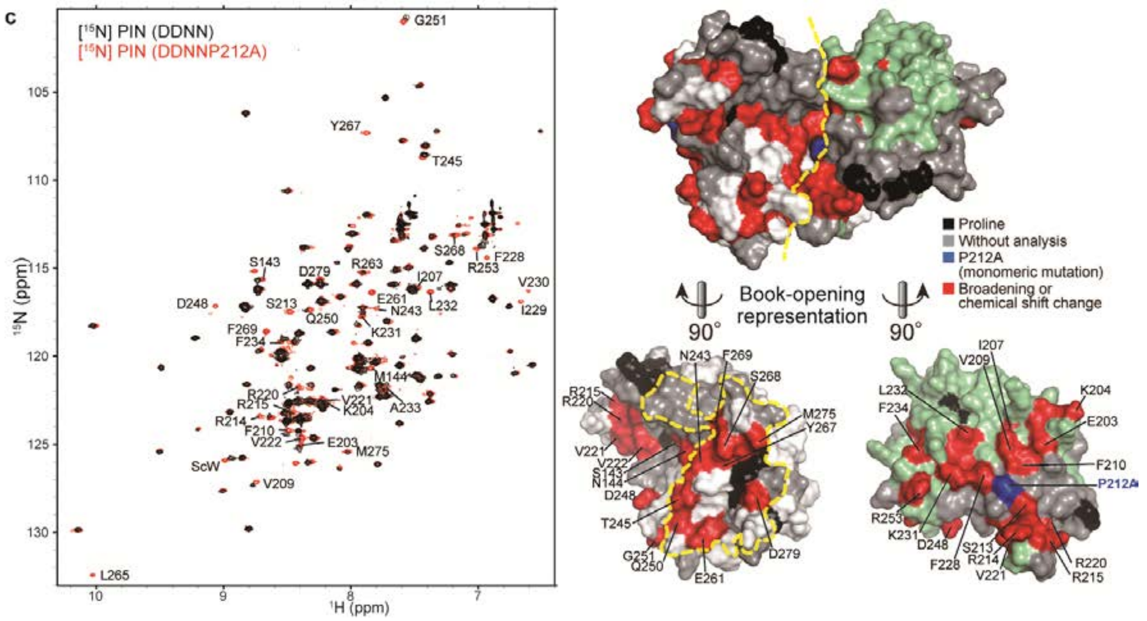

Supplementary Figure 4 NMR analyses of the monomeric PIN mutants. (a) Main-chain resonance assignments of the monomeric PIN mutant (PIN(DDNND278R)) was established by a set of triple resonance measurements using uniformly ^{13}C, ^{15}N double-labeled monomeric PIN mutant, possessing DDNN and D278R mutations. Unassigned and proline residues were colored green and black, respectively, on the ribbon structure of PIN. **(b)** ^{1}H-^{15}N HSQC spectra of

uniformly ^{15}N-labeled PIN(DDNN) and PIN(DDNND278R) were colored black and red, respectively, and superimposed. Signals that exhibited significant broadening or chemical shift changes in PIN(DDNN) were labeled in the spectrum and colored in red on the surface structure of PIN. Each molecule within a PIN dimer was colored white or green. Dimer interface was shown by dotted yellow lines on the dimer as well as the monomer exhibited by book-opening representation. Proline, the residues without analysis, and the monomeric mutation site of D278R were colored black, gray, and blue, respectively. **(c)** ^{1}H-^{15}N HSQC spectra of uniformly ^{15}N-labeled PIN(DDNN) and PIN(DDNNP212A) were colored black and red, respectively, and superimposed. The NMR spectra were analyzed in the same manner as shown in (b).

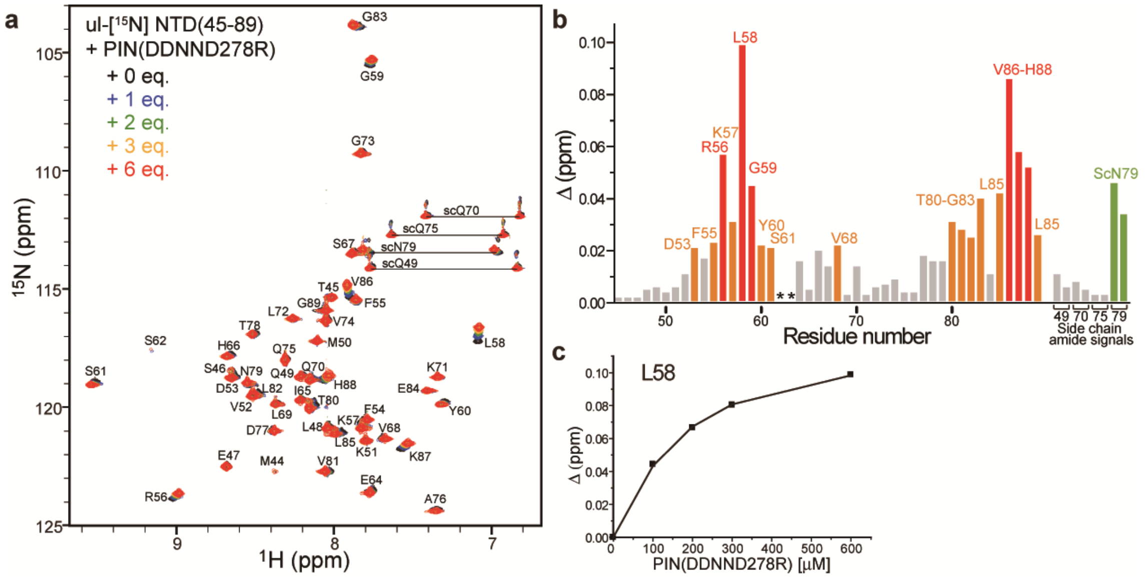

Supplementary Figure 5 NMR analyses of the interaction between NTD and the PIN domain. **(a)** ^{1}H-^{15}N HSQC spectra of uniformly ^{15}N-labeled NTD (residues 45-89, at the concentration of 100 μM) in the presence of 0, 1, 2, 3, and 6 molar equivalents of PIN(DDNND278R) were overlaid in black, blue, green, yellow, and red, respectively. **(b)** The chemical shift difference of each residue upon addition of 6 molar equivalents of PIN(DDNND278R) was calculated using the same equation mentioned in the legend of Supplementary Fig. 2(b). The residues with significant chemical shift changes (Δ(ppm) > 0.045) were colored in red. The residues with 0.02 < Δ(ppm) < 0.045 were colored in orange. The residues without analyses were shown with asterisks. **(c)** Plot and fitting curve of the chemical shift changes in the L58 signal.

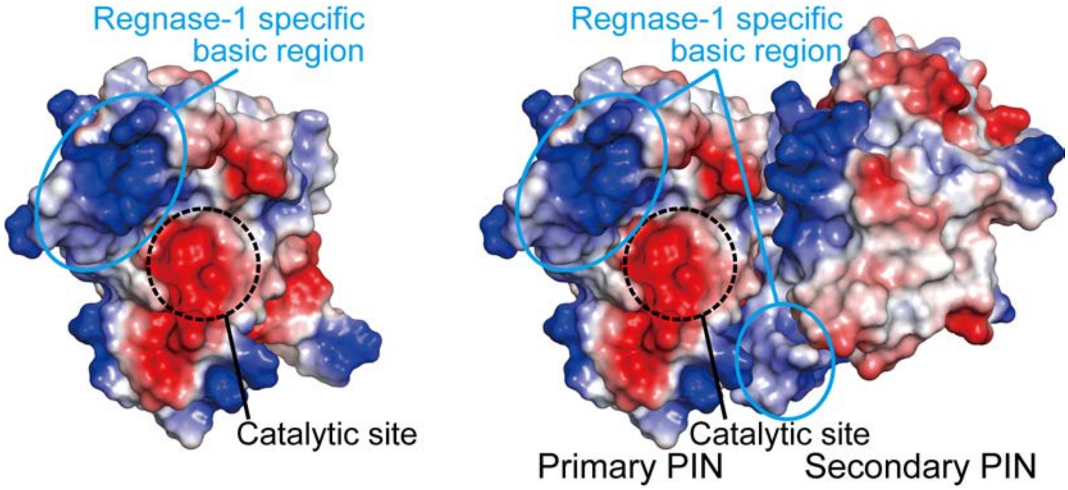

Supplementary Figure 6 Surface potential of monomer (left) and dimer (right) structures of the PIN domain. Catalytic site and Regnase-1 specific basic regions were indicated.

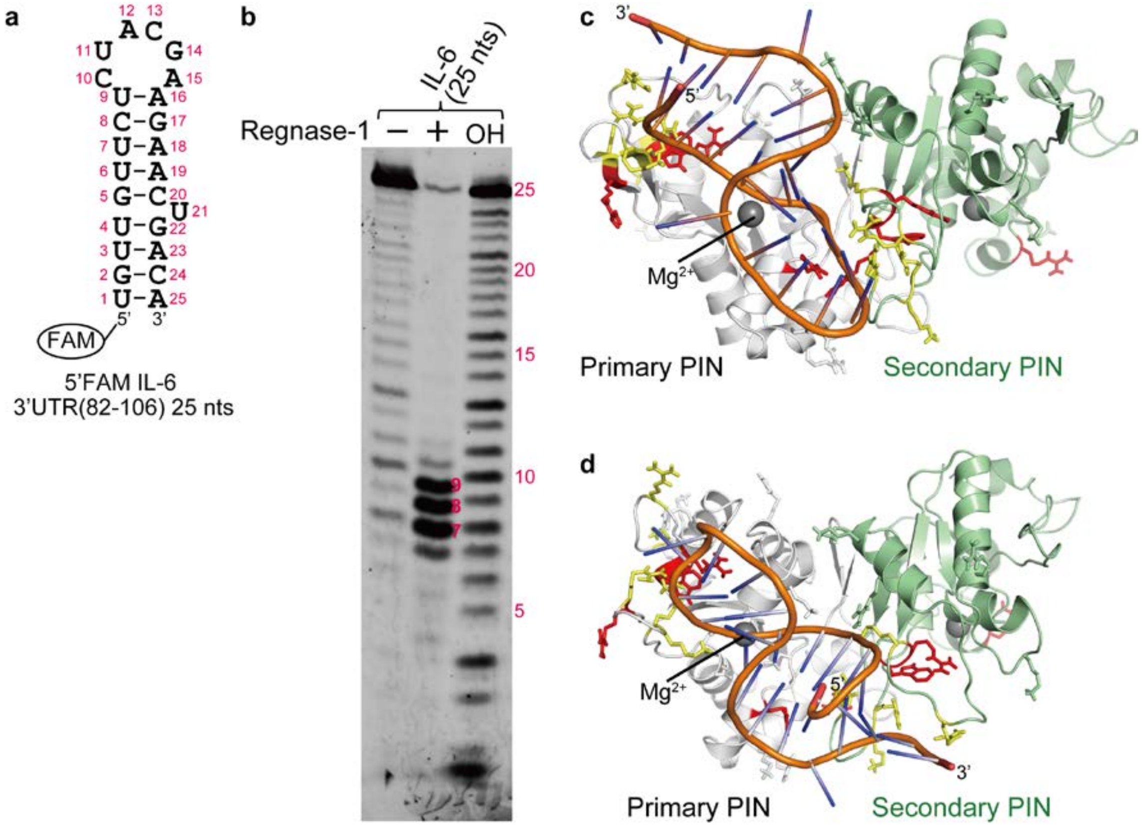

Supplementary Figure 7 Determination of the mRNA cleavage sites by Regnase-1. (a) The sequence and predicted secondary structure of IL-6 mRNA was shown. The numbers indicate the nucleotide chain lengths from 5'-end. **(b)** Fluorescently labeled IL-6 was incubated without (-) and with (+) Regnase-1, or hydrolyzed under the alkaline condition (OH), then samples were applied to denaturing polyacrylamide gel. The cleaved products contained 3'-terminal hydroxyl group while the hydrolyzed RNA contained 3'-terminal phosphate group, so that the mobility of the cleaved RNA fragments is slightly slower than that of the hydrolyzed RNA fragments[1]. **(c)** Docking structure of Regnase-1-IL-6 mRNA 3'UTR. The loop of IL-6 mRNA faces to the primary PIN. **(d)** The direction of the loop region of IL-6 mRNA is opposite

to the model shown in (c). Mutated residues were shown in stick and catalytically important residues were colored red or yellow as in Fig. 4a.

Supplementary Data NMR analyses of the monomeric PIN mutants

We investigated the intermolecular interaction of the PIN domain in solution by NMR. The PIN(DDNN) was not suitable for the assignment of main-chain signals, due to significant broadening of a number of signals. In contrast, the monomeric PIN mutants exhibited NMR signals with uniform intensities. These results indicate that the PIN(DDNN) is in equilibrium between monomer and dimer. Therefore, we applied the PIN(DDNND278R) and established 79% of the main-chain amide resonance assignments of non-proline residues (Supplementary Fig. 4a). The resonance assignments for A146, S148-S156, V179, S181-Q186, R188, D190-V191, I193, G217, C223-R227, Q254, and N271-F274 could not be established. These residues are mainly located in the loop regions and were not assigned probably due to the line-broadening. Based on the main-chain resonance assignment of the PIN(DDNND278R), intermolecular interaction of the PIN(DDNN) was analyzed. The overall spectrum of the PIN(DDNN) was well overlapped with that of the PIN(DDNND278R), indicating that the overall structure of the PIN(DDNND278R) was not largely affected by D278R mutation. A number of residues exhibited significant broadening or chemical shift changes in the PIN(DDNN), indicating that these residues located around D278 to be directly affected by the mutation, and/or were involved in the intermolecular interaction of the PIN(DDNN). In the PIN dimer structure, the affected residues were located not only around the mutated site of D278R,

but also on the opposite surface of the PIN domain that is also involved in the oligomeric interaction in the crystal structure (Supplementary Fig. 4b). P212A is another monomeric mutant that has mutation on the opposite side of D278R-containing oligomeric surface of the PIN domain. Similar to D278R mutant, P212A also exhibited signals with uniform intensities and in comparison with the spectrum of DDNN mutant, not only the residues around P212A mutation but also on the opposite surface of PIN that is involved in the oligomeric interaction in the crystal structure was largely affected (Supplementary Fig. 4c). These NMR results indicate that the PIN domains form a dimer in solution in a similar manner to the crystal structure.

Supplementary Reference

1. Hartmann, R.K., Bindereif, A., Schön, A. & Westhof, E. *Handbook of RNA Biochemistry*, (Wiley, 2015).
